# Hypertrophic Cranial Pachymeningitis and Skull Base Osteomyelitis by Pseudomonas Aeruginosa: Case Report and Review of the Literature

**DOI:** 10.4021/jocmr777w

**Published:** 2012-03-23

**Authors:** Ana Rita Caldas, Mariana Brandao, Filipe Seguro Paula, Elsa Castro, Fatima Farinha, Antonio Marinho

**Affiliations:** a Medicine Department, Santo Antonios’ Hospital, Largo Prof. Abel Salazar, 4099-001 Porto, Portugal

## Abstract

Hypertrophic cranial pachymeningitis (HCP) is an uncommon disorder characterized by localized or diffuse thickening of the dura mater, and it usually presents with multiple cranial neurophaties. It has been associated with a variety of inflammatory, infectious, traumatic, toxic and neoplasic diseases, when no specific cause is found the process is called idiopathic. The infectious cases occur in patients under systemic immunosuppression, which have an evident contiguous source or those who have undergone neurosurgical procedures. We describe a case of a 62-year-old immunosuppressed woman with diabetes and rheumatoid arthritis, which had HCP and osteomyelitis of the skull base caused by pseudomonas aeruginosa, presenting with headache and diplopia. We believe this is the second documented case of pachymeningitis secondary to this microorganism. As a multifactorial disease, it is essencial to determine the specific causative agent of HCP before making treatment decisions, and great care is needed with immunocompromised patients.

**Keywords:**

Pseudomonas aeruginosa; Hypertrophic pachymeningitis; Ophtalmoplegia, optical neuropathy; Osteomyelitis; Skull base

## Introduction

Hipertrophic cranial pachymeningitis (HCP) is a rare clinical entity that is characterized by difuse or localized chronic inflammation and hypertrophy of the dura mater [1, 2]. Recently, there has been an increasing number of HCP reported, probably because of improvements in imaging studies [3]. Several etiologies of HCP have been reported: infectious (bacterial, fungal or viral infections), traumatic, toxic, neoplasic and inflammatory (rheumatoid arthritis, wegener’s granulomatosis, sarcoidosis) [4-6]. Most of the infectious cases occur in patients under systemic immunosuppression, which have an evident contiguous source or those who have undergone neurosurgical procedures [7]. In some cases of HCP, even after an extensive investigation, no specific cause is found, and the process is called idiopathic hypertrophic pachymeningitis or primary pachymeningitis [8].

At the onset of the disease, most of the patients have chronic headache, associated or not with other neurologic manifestations such as cranial nerve palsies (such as ophthalmoplegia, hypoacusia, disphagia), cerebellar ataxia and neuro-ophtamic complications, which include optic neuropathy and even blindness [2, 3, 9]. Cranial gadolinium magnetic resonance imaging (MRI) is more sensitive than enhanced computerized tomography (CT) in demonstrating the dura mater thickening. When there is clinical deterioration or worsening of the neuroimaging despite treatement, a dural biopsy is recommended. The biopsy usually shows dural hypertrophy and chronic inflammation [6].

Skull base ostemyelitis (SBO) typically arises as a complication of malignant external otitis in older male diabetic or immunocompromised patients, and has Pseudomonas aeruginosa as the usual pathogen [10, 11]. Less frequently, skull base osteomyelitis can arise from paranasal sinuses infections or odontic caries [11, 12]; hematogenously spread osteomyelitis is rare in adults [13].

There is only one documented case of HCP and SBO secondary to *Pseudomonas aeruginosa* in the literature [7]. As far as we know, our case is the second documented case.

## Case Report

A 62-year-old woman was admitted to our hospital in late August 2010 with headache and visual complaints which started 2 weeks before.

She had a history of rheumatoid arthritis (RA) since 1991, diabetes mellitus related to chronic corticosteroid therapy since 1995, diabetic retinopathy and chronic venous insufficiency with leg ulceration. She was under chronic corticosteroid therapy (10 mg prednisolone per day for the last year) and insulin therapy. During 2010, she was hospitalized twice due to septic shock related to infected leg ulcers, the first of which in May and the second in July. On both occasions a multi-sensitive *Pseudomonas aeruginosa* was detected in blood cultures and in the wound exudates. On both occasions she was treated with adequate antibiotherapy and had a favorable outcome, with clinical improvement. The blood cultures became sterile on both occasions, two weeks after starting antibiotherapy. After first hospital discharge in June, she remained assymptomatic until 1 week before the second hospitalization, in July. She was then discharged in August, after clinical improvement and with sterile blood cultures.

**Figure 1:**
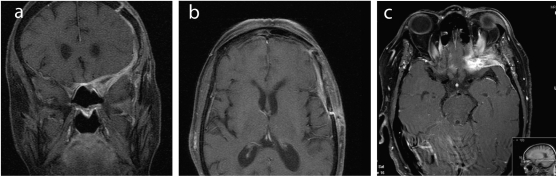
(a-c) MRI images (coronal and horizontal cuts) showing a pachymeningeal thickening around the left anterior clinoid process and lesser wing of the sphenoid, that extends to the left anterior cranial fossa and the anterior part of the middle cranial fossa; it also extends to the orbitary apex through the left optic foramen and superior orbital fissure, affecting the extraocular orbitary muscles, compressing the optic nerve and infiltrating the bone marrow of the lesser wing of the sphenoid and the orbitary wall. This lesion was hypointense on both T1- and T2-weighted images and proeminently enhanced after administration of gadolinium.

A month after the last hospital discharge, she was admitted to our hospital with a 10 day history of an increasing left fronto-orbitary headache and horizontal diplopia; she developed complete left eyelid ptosis a few days before admission. Neurological examination revealed proptosis of the orbit, extraocular ophtalmoplegia (III and IV cranial nerves palsies) and total central amaurosis, all on the left side. She had no other neurologic deficits. Laboratory values were as follows: white blood cells 12.68 × 10^9^ /L (normal range 4.3 - 10.8 × 10^9^/L) with neutrophil predominance, hemoglobin 11.3 g/dL (normal range 12 - 16 g/dL), erythrocyte sedimentation rate (ESR) 88 mm/h (normal range < 10 mm/h), C-reactive protein (CRP) 155 mg/L (normal range < 10 mg/L), angiotensin converting enzyme (ACE) 11 μg/L (normal range 8 - 76 μg/L). The brain MRI (Fig. 1) showed pachymeningeal thickening of the anterior and part of the middle cranial fossa on the left, specially around the anterior clinoid process and the remaining lesser wing of the sphenoid; this thickening extended to the orbitary apex through the left optic foramen and superior orbital fissure, affecting the extraocular orbitary muscles, compressing the optic nerve and infiltrating the bone marrow of the lesser wing of the sphenoid and the orbitary wall; this lesion was hypointense on both T1- and T2-weighted images and proeminently enhanced after administration of gadolinium (Fig. 1). The paranasal sinuses were empty on CT scan. Lumbar puncture showed a clear cerebrospinal fluid (CSF), with normal cell count and biochemical parameters; CSF cultures for bacteria were negative; Enterovirus, Cytomegalovirus, Epstein - Barr, Herpes 6, Herpes simplex 1 and 2 and Varicella-zoster PCR (polymerase chain reaction) testing were negative, as were the *Cryptococcus* count, serologies and immunologic tests. Blood cultures were sterile. Mantoux screening test and quantiferon (IGRA - interferon-γ release assay) were negative. Immunophenotyping of the peripheral blood was normal. The immunologic study showed increased rheumatoid factor (551 UI/mL) and anti-CCP antibodies (1471 U/mL), which were similar to previous known results. The otological exam and orthodontic examination were normal.

Considering the clinical picture, the diagnostic tests and the patient’s known diseases, the possibility of an inflammatory or granulomatous lesion was placed. The patient was treated with a high dose of intravenous methylprednisolone (1g per day during 5 days), followed by 40 mg of prednisolone per day (tapering after 1 week), but she got worse, developing right eyelid ptosis and total bilateral amaurosis; the MRI showed no change. The bad clinical response to the high-dose of corticosteroid and the immunocompromised state of the patient raised suspition of a fungal or bacterial infection, so we started amphotericin B plus ceftazidime (this antibiotic was started because of the suspicion of an infection caused by Pseudomonas aeruginosa, based on the previous blood cultures of the patient).

A brain biopsy was scheduled and it showed a thickened dura mater, chronic non-granulomatous inflammation compatible with osteomyelitis and tissue predominance of gram negative bacteria, identifyed as *Pseudomonas aeruginosa*, similar to the one identified on both blood cultures performed a few months before. We stopped amphotericin B and kept ceftazidime (2 g every 8 hours), according to the bacteria sensitivity. As osteomyelitis was present, we decided to do a 12-week course of antibiotherapy during hospital stay, with good clinical response. The headache only improved with opioids. There was an improvement of the ocular and eyelid movements, but her vision did not improve, so that she maintained total bilateral amaurosis, despite the antibiotherapy. Visual evoked potentials showed a serious compromise of the pre-chiasmatic component, probably irreversible. Beyond the neurologic deficits, she showed clinical improvement, with no other clinical problem.

In spite of this clinical improvement, the MRI performed at the end of the 12 weeks revealed the same lesion, now with partial occlusion of the internal carotid artery. The whole-body FDG-PET (fluorodeoxyglucose-positron emission tomography) showed no hypermetabolic activity in the brain, which led us to interpret the MRI lesion as fibrosis.

During her hospital stay we provided occupational therapy to begin her adaptation to her new life.

When the patient was discharged she was asymptomatic; she was under prednisolone 10 mg per day. After discharge, she was followed in regular medical consultations, and two months after hospital discharge she maintained the previously described neurological deficits, otherwise asymptomatic, with no signs of infectious relapse.

## Discussion

HCP refers to a well-circumscribed area of chronic inflammatory dura thickening, but all three meningeal layers become fused by dense fibrotic membranes [7]. There is no sex or age predominance [14]. Patients usually present with multiple progressive cranial neuropathies [5]; the clinical features appear to be associated with the location of the abnormal dura mater observed on gadolinium MRI [6]. The optic nerve is the most frequently affected [5], and when it is envolved, the visual prognosis is poor [15]. MRI imaging tipically reveals well-circumscribed hyperintense laminar thickening of the dura, with low T2 signal intensity and enhancement after intravenous infusion of gadolinium or a similar agent [16, 17].

HCP may be primary or idiophatic when no cause is found, or secondary when identifiable causes exist [18]. The most frequent causes of secondary HCP are infectious; syphilis and tuberculosis are the most frequently reported, mainly in developing countries or in immunocompromised patients [19]. Other pathogens such as Candida, Aspergillus, Pseudomonas or any other that cause infection of the extra or subdural space, paranasal sinuses or mastoid, can induce thickening of the dura mater [20-23]. Non-infectious causes of secondary HCP include traumatic, inflammatory (sarcoidosis, rheumatoid arthritis, Wegener’s granulomatosis, poliartreitis nodosa, among others) and toxic causes [4, 7, 14].

HCP presents with symptoms that can mimic other diseases, such as subdural hematoma [24, 25] or dural carcinomatosis [26], so that some imagiologic exams have to be done to exclude those clinical entities.

This rare disorder is a diagnosis of exclusion, because its appearance on MRI is often similar to other diseases as meningioma en plaque, lymphoma, tuberculosis and sarcoidosis; when the systemic workup is nondiagnostic, craniotomy with meningeal biopsy is required to differenciate between those diseases [7, 17]. When we have a typical image on the MRI and typical histologic findings, we should seek the cause of HCP, if not found, it´s called idiopathic HCP or primary HCP; idiopathic HCP is always a diagnosis of exclusion [14, 27].

The first case of pachymeningitis was reported in 1869 by Charcot and Joffroy [28]; since then, there have been several cases reported, secondary to a variety of causes, however, most cases remain idiopathic [9, 26].

Most of the cases of infectious HCP are related to bacterial sphenoid and ethmoid sinusitis and chronic otitis media [29, 30]; the hematogeneous spread of infections is not common in HCP, and there are no cases described on the literature. On the other hand, most of the cases of skull base osteomyelitis originate in malignant external otitis, less frequently in paranasal sinusitis and rarely from hematogeneous spread [31, 32].

In this case, our patient had HCP and osteomyelitis of the skull base, probably caused by *Pseudomonas aeruginosa*, the only isolated bacteria. Before the results of the biopsy, the bad initial response to corticotherapy was a clue to an infectious cause, either fungal or bacterial; furthermore, our patient was diabetic and immunocompromised due to chronic corticotherapy, which implies greater susceptibility to infections. In addition to the suggestive findings of HCP and osteomyelitis on the MRI, her clinical picture was typical and the biopsy findings strongly suggested those diagnostics. Initially there was no clear route of entry for the microorganism; we did an extensive imagiologic and clinical evaluation for a contiguous source, but there was no evidence of infeccious sinusitis, external or media otitis or other kind of contiguous infection. *Pseudomonas aeruginosa* was the only bacteria identified in the biopsy specimen and in the cultured tissue sample, similar to the one identified on both blood cultures performed a few months before; we assumed therefore that our patient had hipertrophic cranial pachymeningitis and skull base osteomyelitis caused by *Pseudomonas aeruginosa*, which probably resulted from a hematogenous spread.

According to the literature, meningitis caused by *Pseudomonas aeruginosa* is rare [33], and it’s more common in immunocompromised patients, who have undergone neurossurgical procedures [34] or who have chronic otitis media [35]. Skull base osteomyelitis caused by *Pseudomonas aeruginosa* is generally caused by malignant external otitis, and it has also been described as arising from infection f the paranasal sinuses and from mandible or maxilla due to odontic caries [36, 37]. Taking into account that our patient had two previous episodes of bacteriemia (from infected venous ulcers of the leg) due to the same strain of *Pseudomonas aeruginosa* found in the brain biopsy, this was the more obvious route of entry for the infection, although rare. As far as we know, this is the second case of *Pseudomonas aeruginosa* HCP and skull base osteomyelitis described in the literature; the first one was an immunocompetent 60-year-old man, described by Girkin CA et al in 1998 [7].

Our patient neurologic deficits improved with the 12-weeks course of ceftazidime, in spite of the persistent bilateral amaurosis, which was probably due to irreversible optic nerve destruction. The overall good response to antibiotherapy helped to confirm the infectious etiology of this HCP. Infectious cases are not well reported in the literature, but there is a high mortality risk; a high index of suspicion may be life saving. Involvement of the optic nerve usually confers a poor visual prognosis. Antimicrobial therapy may show some visual improvement in the infectious cases; in non-infectious cases some improvement in visual acuity has been shown with high dose corticosteroid treatment [38]. Some cases recover after surgical removal, but the disease progression is always uncertain, and relapses may occur [26].

Cranial gadolinium MRI is more sensitive than contrast enhanced CT in demonstrating the thickened abnormal enhancing dura-mater; non-contrast CT should be used to exclude neoplasia [39]. The definitive diagnosis is made by histophatological findings, which usually show an inflammatory process with lymphomononuclear cell infiltration and a thickened dura-mater characterized by a dense hypocellular fibrous tissue [1, 40]. In relation to the follow-up of the disease there is no stablished consensus. On this particular case, we had a persistent pachymeningeal thickening on the MRI, which did not improve after the 12-week course of antibiotherapy; the clinical improvement of the patient and the absence of hypermethabolic lesions on the PET scan were the facts that led us to consider that the infectious/inflammatory lesion was improving. There are some studies showing the role of gallium-67 scintigraphy and FDG-PET on the follow-up and detection of relapse of HCP, not possible with the MRI [41-43]. Various nuclear medicine imaging techniques have been advocated in suspected SBO, including gallium-67 scintigraphy, white blood cell scans, technetium-99 bone scans, and PET (positron emission technique) scans [12, 44]. The latter two techniques may have an advantage over CT and MRI in revealing persistent osteomyelitis and may be more useful in the follow-up of patients under antibiotics, because marrow signal change may persist for up to 6 months after successful treatment. There is currently no imaging technique to replace the role of biopsy for microbiological culture in cases of suspected SBO [11, 44].

There is also no consensus about the length of treatment of HCP and SBO. There is not a minimal time of antibiotherapy advised in the case of infectious HCP. The length of antibiotherapy in SBO is variable among the cases reported, but globally treatment was given for at least 1 month and up to 6 months [11, 12, 44]. Adjuvant hyperbaric oxygen may also provide benefit [12, 44]. We decided to do a 12-week course of antibiotherapy to treat our patient, having into account the improvement of the symptoms and the imaging findings in the PET scan.

Most of cases of HCP are idiopathic, and they have a good response to steroids [45, 46]. Most of the patients treated with them show improvement and stabilization, which gives a clue about the inflammatory nature of most of these lesions [46, 47]. In some cases, due to the need of large doses of corticosteroids, these patients are also treated with immunomodulators, like methotrexate or azathioprine [26, 48-50]. Non-idiophatic HCP, like infectious or malignant conditions, is not responsive to corticosteroids but are usually responsive to specific therapies, as antibiotics or radiotherapy [51]. The surgical treatment is rarely used in HCP, although surgical optic channel decompression [45, 48] and ventriculo-peritoneal derivation [52] are sometimes necessary in cases of obstructive hydrocephaly.

Due to the extensive differential diagnosis of this clinical entity, the evaluation of HCP requires blood and cerebrospinal fluid (CSF) evaluation, contrast MRI, a physical examination and in most cases a dura mater biopsy. Serum angiotensin converting enzyme, c and p-ANCA, rheumatoid factor and anti-nuclear antibody must be tested; an evaluation of Lyme, syphilis, tuberculosis and fungi is also necessary [8, 14]. The CSF findings are dependent on the etiology; when infection is not present, CSF frequently shows non-specific pleocytosis, predominantly lymphocytic; there may be an increase in protein content [39, 51].

Systemic autoimmune and rheumatologic diseases that can present with similar clinical and radiologic features must be considered in the evaluation of HCP; for example, patients with RA sometimes develop headache and optic neuropathy with fibrosis and chronic inflammatory infiltrate of the dura-mater [53, 54]. In our patient´s case, she had RA and had a chronic inflammatory infiltrate of the dura-mater, but it was proved to be infectious at the end; as she was an immunocompromised patient, the infectious cause should have been considered since the beginning.

Biopsy of the dural lesion should be considered and it’s recommended when the patient clinically deteriorates or the neuroimaging worsens despite treatment; it’s a low risk procedure, and it’s considered the gold-standard for the diagnosis [48, 52]. Chronic inflammation of the usually inert dura-mater with minimal or no inflammation of pia and arachnoid should be seen in HCP [6, 48].

The natural course of this disease isn´t completely known. HCP frequently progresses if untreated; the idiopathic cases treated with corticosteroids usually improve, but that benefit may be temporary and partial [9, 55]. In some cases there is a subclinical progression of the disease despite long-term treatment [55]. The optic neuropathy usually improves or stabilizes; the majority of patients with visual loss maintain their vision because it is usually secondary to inflammation rather than compressive fibrosis around the optic nerve [6]. Our patient had permanent visual loss, probably due to compressive fibrosis, as described above.

Oclusion of the intracranial vessels by the inflammatory mass is a frequent complication of HCP, namely the internal carotid artery and dural sinuses [48, 56]. It happened on our case, but apparently our patient remained clinically stable, with no new neurologic deficits.

There are some authors that speculate that inflammatory myofibroblasyic tumor (IMT), Tolosa-Hunt syndrome (THS) and idiopathic HCP may be various presentations of a spectrum of inflammatory disorders that have diverse locations but share similar histologic, clinical and imaging findings [57, 58]. IMT is also known as “myofibroblastoma” or “inflammatory pseudotumor”, and is characterized by proliferation of myofibroblastic spindle cells with mixed inflammatory infiltrates of plasma cells [59]; IMTs often include orbital involvement [60]. THS is a retro-orbital pseudotumor that involves the cavernous sinus, with imaging findings similar to IMT [39, 58]; it causes a painfull oftalmoplegia due to a nonspecific granulomatous inflammation of the cavernous sinus. Some described cases of THS have concurrent pachymeningitis in other portions of the cranial vault, raising the possibility that THS may be a focal manifestation of idiopathic HCP [61]. On the other hand, there are cases of idiopathic HCP with a simultaneous orbital mass [62]. The main clinical features of these 3 disorders include headache and cranial polyneuropathy, usually leading to visual symptoms [57, 61]; they all typically respond to steroid therapy [61]. This reinforces the idea that these clinical entities may somehow be related and may possibly be different presentations of the same disease [63]. This comparison can only be made between IMT, THS and idiopathic HCP, since infectious HCP is a different entity and needs another clinical approach, as was the case of our patient.

In conclusion, HCP is a unique clinical entity characterized by fibrosis and thickening of the dura mater and variable neurological disfunction. However, there is a wide variety of causes of HCP – infectious, granulomatous, neoplasic, and inflammatory. There are even authors that argue that this is not a single disease but a wide variety of diseases with similar clinical expression but with different therapeutic approach. There is no consensus on the literature about the most adequate treatment of this disease, probably because of the wide variety of diseases we are talking about and the rarity of the disease.

Before making decisions about the most adequate treatment, it’s essencial to determine the specific etiology of the HCP; corticosteroids must be used carefully, mainly in patients with history of tuberculosis or the immunocompromised, as we had in the case described; infectious causes must always be excluded. On this particular case, with the known history of bacteriemia due to Pseudomonas aeruginosa, the hipotesis of infectious HCP should have been put sooner. In fact, it seems that an adequate and prompt treatment of HCP is the main key of the therapeuthic success of these patients.

There is still a lot to clarify about this subject in the literature. There is a lack of complete follow-up throughout the progress of the disease; there are only sporadic cases, and the majority is labeled as idiopathic. A lot of studies are still needed to understand this clinical entity.
